# Spatial Distribution and Ecological Assessment of Vegetation–Climate Zone Ecotones in the Temperate Forests of South Korea

**DOI:** 10.1002/ece3.74306

**Published:** 2026-09-06

**Authors:** Byeong‐Joo Park, Eun‐Seo Lee, Kwangil Cheon

**Affiliations:** ^1^ Baekdudaegan National Arboretum Bonghwa Republic of Korea; ^2^ Carbon Sequestration Research Team National Institute of Ecology Seocheon Republic of Korea; ^3^ Ecosystem Service Team National Institute of Ecology Seocheon Republic of Korea

**Keywords:** ecotone, protected area, South Korea, species turnover, vegetation–climate zone, zeta‐diversity

## Abstract

Ecotones are transition zones that integrate environmental gradients and play an important role in biodiversity conservation; however, within the context of South Korea, their ecological functions and spatial characteristics remain insufficiently quantified at the national scale. Scientific evidence is needed to support their inclusion in protected area planning. In this study, we quantified the spatial distribution and ecological characteristics of ecotone zones (EZs) across vegetation–climate zones in the temperate forests of South Korea and examined their associated biodiversity patterns and species turnover. Entropy‐based spatial classification was conducted to delineate the EZs. Environmental and compositional differences among zones were tested using ANOVA and multivariate ordination (CCA, MRPP), while species turnover and community‐assembly processes were examined through zeta‐diversity–based model comparisons (RMSE, AIC). The EZs exhibited mixed characteristics of multiple vegetation–climate zones, showing intermediate environmental conditions and transitional species composition consistent with a buffering role. EZs showed high species richness (607.8 ± 9.6 species per grid) that did not differ significantly from the Northern Temperate Zone (NTZ) (*p* = 0.069), and CCA explained the compositional gradient, with the first two axes accounting for 86.5% of the constrained variance (CCA1 = 57.2%, CCA2 = 29.3%). MRPP results indicated the greatest compositional overlap between EZ and the Central Temperate Zone (A = 0.0317), the lowest agreement value among all pairwise comparisons. The zeta‐diversity analysis revealed unimodal patterns with intermediate turnover and partial stability dominated by stochastic processes. These patterns indicate that EZs are transition zones associated with high species richness and intermediate turnover. Overall, our findings suggest that EZs may warrant consideration in protected‐area prioritization and provide a quantitative basis for identifying them at the national scale.

## Graphical abstract

Using a national 800‐grid framework, we classified vegetation–climate zones in South Korea into ecotone and core areas based on threshold values derived from zonal statistics. We then integrated species occurrence, climate, topography, and ecosystem service variables to compare biodiversity and ecological characteristics among these zones.

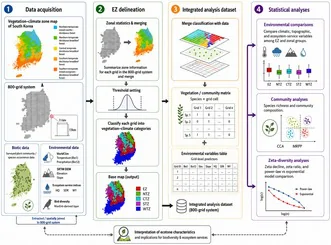

## 1 Introduction

Global biodiversity policy increasingly emphasizes not only the quantitative expansion of protected areas but also their ecologically informed selection. The Kunming–Montreal Global Biodiversity Framework (KMGBF) sets a target of protecting at least 30% of land and sea area (Convention on Biological Diversity 2024), which highlights the need for scientific evidence to guide where new protected areas should be located (Park et al. 2024). In South Korea, protected areas are typically designated based on habitats of endangered species or specific ecosystem types. Biodiversity‐related criteria include regions containing rare or endemic plants, representative ecosystems, or forest genetic resources. As of 2023, protected areas accounted for approximately 19.1% of South Korea's land area. To achieve KMGBF Target 3, the government is increasing the designation of new protected areas, particularly in regions with outstanding biodiversity (Ministry of Environment 2023). However, beyond quantitative expansion, a new paradigm for selecting protected areas based on ecological evidence is needed. This expansion also necessitates a shift toward ecologically evidence‐based selection criteria, including the systematic consideration of ecotone zones (Choe et al. 2018).

In this context, ecotone zones (EZ) are an ecological concept worthy of attention. EZ are defined as transition zones formed between two adjacent ecosystems or vegetation zones (Odum 1971). These areas are characterized by overlaps in habitats or vegetation–climate zones, where diverse species and functional traits coexist. As a result, EZ serve as repositories of biodiversity and genetic diversity and are considered key ecological areas for native species (Odum 1971). More recently, Hufkens et al. (2009) redefined ecotones as “a multi‐dimensional environmentally stochastic interaction zone between ecological systems with characteristics defined in space and time, and by the strength of the interaction,” emphasizing their dynamic and spatially heterogeneous nature. This definition aligns with the entropy‐based spatial delineation approach employed in the present study. In the mid‐latitude context of East Asia, ecotones at vegetation–climate boundaries represent particularly dynamic and understudied systems (Moon et al. 2017).

The mid‐domain effect (MDE) predicts that species richness peaks at intermediate positions within a bounded geographic domain due to geometric constraints on species ranges (Colwell and Lees 2000). In the context of vegetation–climate ecotones, this theoretical framework predicts that transition zones occupying intermediate positions along climatic gradients may exhibit elevated species richness, independent of environmental productivity. Rahbek (1995) and Grytnes (2003) observed similar hump‐shaped richness patterns along altitudinal gradients, lending empirical support to the MDE. Testing MDE predictions in the context of national‐scale EZ classification is therefore of both theoretical and applied significance.

EZ should be understood not merely as spatial boundaries but as dynamic ecosystem elements reflecting organisms' responses to environmental change (Risser 1995), with soil characteristics often differing from surrounding ecosystems and influencing vegetation structure and diversity patterns (Marfo et al. 2018). Across diverse ecosystems, previous studies have constructed EZ databases through ecogeographic analyses incorporating species diversity, climate‐driven migration, soil environments, vegetation changes, and vegetation–climate zone transitions (Veblen and Lorenz 1988; Gottfried et al. 1998; Marimon et al. 2006; Zeng et al. 2007; Virtanen et al. 2010; D'Odorico et al. 2013). However, EZ responses are not always dynamic or unidirectional: some studies report relatively stable EZ locations and structures rather than directional shifts (Tölgyesi et al. 2016), while dispersal limitation and stochastic processes near restoration sites can produce uncertain species compositions (Peringer and Rosenthal 2011). These contrasting findings indicate that EZ dynamics vary with spatial constraints and stochastic factors, underscoring the need for quantitative analyses supported by long‐term monitoring (De Klerk et al. 2018).

Classical treatments of ecotones (Odum 1971; Risser 1995) conceptualize them at the scale of individual ecosystems, where a boundary is drawn between two adjacent, relatively homogeneous communities. In this study, we adopt a landscape‐ecological perspective, in which the unit of analysis is not a single boundary line but a landscape mosaic—the standard national survey grid—within which the co‐occurrence of multiple vegetation–climate zone patches indicate a higher probability of ecotone formation (Collinge 2009). By linking this landscape‐scale framework to a nationwide survey grid, we identify cells with a high likelihood of harboring ecotonal structure, rather than delineating the precise spatial extent of individual ecotone boundaries. This distinction matters ecologically: landscapes with a greater diversity of co‐occurring vegetation–climate patches have been argued to support elevated biodiversity through habitat heterogeneity and connectivity among patch types (Ries et al. 2004; Kark 2012), consistent with the elevated species richness we observe in EZ (Section 3.3). Framing EZ as high‐probability ecotone landscapes therefore allows the national‐scale screening of areas where landscape heterogeneity—and its associated biodiversity value—is likely to be concentrated, providing a practical tool for conservation prioritization rather than a fine‐grained ecotone map.

In this study, plant distribution data from the Korean Nationwide Survey on Natural Environments were combined with vegetation–climate zone data from the Korea National Arboretum to quantitatively analyze biodiversity relationships between overlapping vegetation–climate zones and core vegetation–climate zones.

South Korea has a monsoon climate (Park, Kim, et al. 2019), and seasonal rainfall variability can enhance biodiversity by creating environmental heterogeneity that supports diverse vegetation types and species (Veblen and Lorenz 1988; Marimon et al. 2006). Based on this climatic context, this study assumes that EZ exhibit dynamic biodiversity patterns. The following hypotheses were tested:

*EZ will show significantly different distributions of environmental variables compared to core vegetation–climate zones*.

*Species composition in EZ will overlap with species composition in each core vegetation–climate zone*.

*EZ will show intermediate species turnover patterns most similar to CTZ, reflecting their transitional position between NTZ and CTZ along the temperature‐altitude gradient, with stochastic processes partially governing community assembly* (Chase and Myers 2011; Vellend 2010). *Despite growing recognition of ecotones' ecological importance, no consistent, quantitative method exists for identifying landscape‐scale vegetation–climate transition zones across a national territory. Addressing this gap, we aimed to* (*i*) *delineate EZ objectively at the national scale using an entropy‐based classification, (ii) characterize their environmental conditions, species composition, and turnover relative to core zones, and (iii) thereby provide a quantitative, evidence‐based foundation for considering EZ in protected‐area planning*.

## 2 Materials and Methods

### 2.1 Threshold Setting and EZ Categorization

To categorize EZ, raster data (pixel size = 30 m × 30 m) for five vegetation–climate zones designated by the Korean National Arboretum were used: northern temperate mixed coniferous–deciduous forest, northern temperate deciduous broadleaf forest, central temperate deciduous broadleaf forest, southern temperate deciduous broadleaf forest, and southern temperate mixed evergreen–deciduous broadleaf forest. These data were combined with vector data dividing South Korea into 800 grid cells (11 km × 13 km each) (Korea National Arboretum 2020).

The grid system was derived from a 1:25,000 digital topographic map and served as the standard spatial unit for the Ministry of Environment's Nationwide Survey on Natural Environments (National Institute of Ecology 2019).

The two GIS datasets were overlaid, and the Zonal Statistics as Table tool in ArcGIS Pro (v3.5, ESRI, Redlands, CA) was used to calculate the relative area (pixel proportion) of each vegetation–climate zone within each grid cell. Cells were classified as core vegetation–climate zones when the pixel proportion of a single zone exceeded a predefined threshold. Cells in which no vegetation–climate zone exceeded the threshold were defined as EZ.

Thresholds from 50% to 70% were tested, and 70% was selected as the final threshold. At this threshold, a cell was classified as a core zone when a single vegetation–climate zone occupied ≥ 70% of its area, and as an EZ otherwise. The 70% threshold was selected because it maximized the entropy difference between EZ and core zones (Table S1) while representing homogeneous core regions (Hufkens et al. 2009; Rocchini et al. 2013). The Shannon and Simpson entropy indices were calculated from the proportional coverage of vegetation–climate zones within each cell to characterize compositional heterogeneity. Because these indices are derived from the same proportions used to delineate EZ, we did not treat them as independent validation; instead, we assessed the robustness and ecological validity of the classification through three additional analyses: a threshold‐sensitivity analysis (Table S1), a grid‐size sensitivity analysis (Table S2), and validation against independent species‐distribution data not used in the delineation (Table S3). Shannon entropy was normalized by its maximum value to represent heterogeneity or mixing within the cell (Shannon 1948). The normalized Simpson index (Simpson 1949) estimates the likelihood that two randomly selected pixels belong to different vegetation–climate zones, thereby quantifying compositional diversity (Jost 2006; Rocchini et al. 2013; Feilhauer et al. 2021). Both indices are widely used in ecology to assess diversity.

Although originally developed to measure species diversity, these indices were applied here to quantify heterogeneity in vegetation–climate zone composition within grid cells (Ricotta 2005; Rocchini and Ricotta 2007). This approach represents a generalized application of “ecological entropy” and is useful for evaluating compositional heterogeneity (Paz‐Kagan et al. 2017).

When the proportional pixel count for each vegetation–climate zone within a grid cell is defined as *pi*, the Shannon entropy (H') and Simpson indices(D) are calculated as follows:

$$H^{\prime }=-\sum p_{i}\ln\left(p_{i}\right),D=1-\sum p_{i}^{2}$$

where the Shannon entropy reflects the degree of heterogeneity, while the Simpson index reflects dominance within a grid cell. The interpretation of index values varies according to the magnitude of entropy.

The threshold that maximized differences in entropy between EZ and core vegetation–climate zones was selected as the optimal boundary. This approach is consistent with the ecological concept of defining EZ as “transition zones that maximize heterogeneity between adjacent ecosystems” (Odum 1971; Risser 1995; Hufkens et al. 2009). The optimal boundary is the threshold at which differences between the two indices are maximized and, in ecological terms, represents a distinct classification boundary (Collinge 2009; Hufkens et al. 2009).

To evaluate whether EZ delineation was sensitive to methodological choices, we conducted two sensitivity analyses. First, we varied the classification threshold from 50% to 70% and compared the resulting entropy separation between EZ and core zones (Table S1). Second, we re‐aggregated the vegetation–climate zone raster at grid sizes from 0.25× to 4× the original survey unit and repeated the classification (Table S2). To address the potential circularity between the entropy‐based delineation and entropy‐based description, we further validated the classification using independent species‐distribution data—endemic, endangered, alien, and total species richness—that were not used in the entropy calculation (Table S3). Differences among zones were tested using Kruskal–Wallis tests.

In this study, the Northern temperate mixed coniferous–deciduous forest occupied a very narrow area and was therefore combined with the Northern temperate deciduous forest for analysis. Thus, a total of five groups were classified: four vegetation‐climate zones and EZ.

### 2.2 Comparison of Environmental and Ecological Factors

Each grid cell was treated as an individual survey plot, and plant species presence/absence data were extracted from the Nationwide Survey on Natural Environments. Species distributions were organized by grid cell using the Spatial Join function of ArcGIS Pro. The survey period spanned 2006–2023.

**FIGURE 1:**
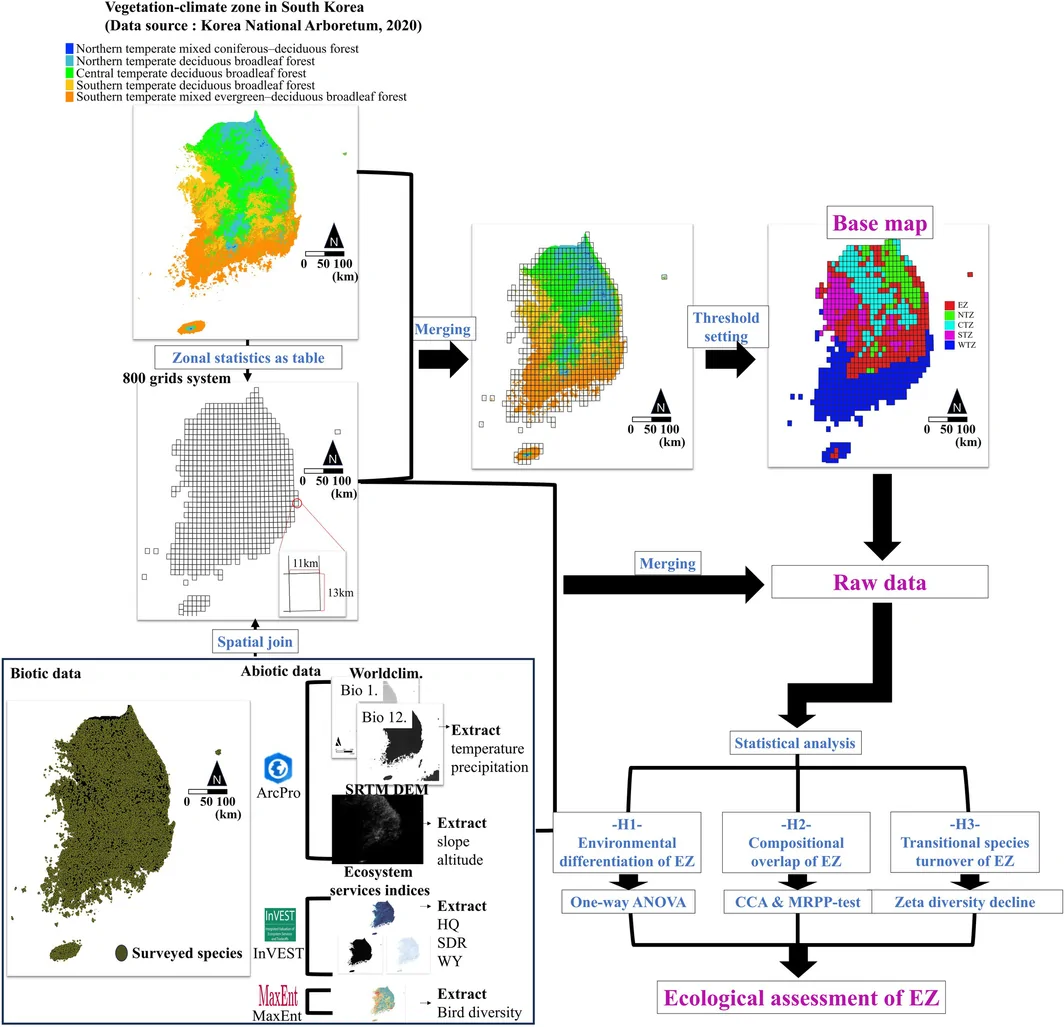
Overall workflow of the study. Biotic and abiotic data were spatially joined to an 800‐grid system. Vegetation–climate zones were classified using an entropy‐based threshold to delineate ecotone zones (EZ) and produce the base map. From the integrated dataset, three hypothesis‐specific analyses were performed—environmental differentiation among zones (H1, one‐way ANOVA), species composition (H2, CCA and MRPP test), and species turnover (H3, zeta‐diversity)—which together informed the ecological assessment of EZ.

Environmental variables extracted for the 800 grid cells included temperature, precipitation, altitude, slope, habitat quality (HQ), bird diversity, water yield (WY), and sediment delivery ratio (SDR) (Figure 1). These variables have been identified in previous studies as major climate and topographic factors influencing plant distribution and ecological characteristics (Oliver and Larson 1990; Wilson 1992; Kimmins 1997; Thuiller et al. 2005; Sekercioglu 2006; Körner 2007; Lavorel et al. 2008; Maestre et al. 2012).

WorldClim Bioclim data (v2.1; 30 arc‐s resolution; mean values for 1970–2000) were used for temperature and precipitation. These 30‐year climatic normals were used to represent the long‐term spatial climatic gradient to which vegetation–climate zones and plant community distributions respond, rather than the weather conditions of any specific survey year. The offset between this reference period and the survey period (2006–2023) is addressed in the Discussion. A digital elevation model from NASA's Shuttle Radar Topography Mission (SRTM; 30 m resolution) was used to derive altitude and slope.

Three ecosystem service indices (HQ, WY, and SDR) were applied based on plant species distributions across ecological niches following National Institute of Ecology (2022). These indices were calculated using InVEST (v3.14.1; Stanford University, CA, USA) (National Institute of Ecology 2022). The input datasets (with their sources and reference periods) and all model parameter settings—including LULC classification, threat layers, sensitivity scores, and biophysical coefficients—are provided in Table S4. Applying ecosystem service indices to species composition analysis enables multifaceted socio‐ecological interpretations (Park and Cheon 2025).

HQ was calculated by considering habitat type, habitat sensitivity, and threat level. Habitat type was determined using Land Use and Land Cover (LULC) data. HQ serves as a supporting index for qualitative assessment of biodiversity (Xu et al. 2019) and is expressed on a scale from 0 and 1, with values closer to 1 indicating conditions more favorable for ecosystem conservation. It was calculated using the equation provided by the National Institute of Ecology (2022).

(1) $$\mathrm{HQ}=H\times \left(1-\left(\frac{D^{2}}{D^{2}+K^{2}}\right)\right)$$

HQ: Habitat quality.

H: Habitat suitability based on land use type.

D: Threat level per habitat type.

K: Half‐saturation constant.

Bird diversity was estimated using a maximum‐entropy (MaxEnt) species‐distribution model based on bird occurrence data from the Third Nationwide Survey on Natural Environments (87 species), following the standard protocol of the National Institute of Ecology (2022). It was therefore treated as a survey‐based biodiversity variable derived from a species‐distribution model, distinct from the InVEST‐derived ecosystem‐service indices (HQ, WY, and SDR). WY was calculated by subtracting mean annual actual evapotranspiration (AET) from mean annual precipitation for each grid cell, according to the following equation (National Institute of Ecology 2022).

(2) $$\mathrm{WY}=\left(1-\frac{\mathrm{AET}}{\mathrm{PRE}}\right)\times P$$

where WY is water yield, AET is annual actual evapotranspiration, and PRE is annual precipitation.

SDR was calculated based on precipitation‐ and erosion‐related factors and slope variables, according to the following equation (National Institute of Ecology 2022).

(3) SDR=R×Ks×LS×C×P

where SDR is sediment delivery ratio, R is rainfall erosivity factor, K_s_ is the soil erodibility factor, LS is the slope length and steepness factor, C is the cover management factor, and P is the support practice factor.

One‐way analysis of variance (ANOVA) was used to compare mean values among each group. Results are presented as mean ± standard error (SE), and significant differences among vegetation–climate zones were identified using Tukey's HSD post hoc test (*p* < 0.05).

### 2.3 Species Composition Analysis

To compare alpha diversity among groups, species richness was calculated as the number of species recorded in each grid cell (Magurran 2004). One‐way ANOVA followed by Tukey's HSD post hoc test was used to compare species richness among groups (*p* < 0.05).

To analyze relationships between vegetation–climate zones and environmental variables, canonical correspondence analysis (CCA) and multi‐response permutation procedures (MRPP) were performed using PC‐ORD (v7.0, MjM Software Design, Gleneden Beach, OR, USA). CCA is a multivariate method used to visualize and interpret the effects of environmental variables on differences in species composition among vegetation–climate zones and to compare species composition and distribution between groups (McCune et al. 2002). A presence–absence matrix was used as the primary matrix, and a second matrix was constructed from the eight environmental variables (temperature, precipitation, slope, elevation, HQ, bird diversity, WY, and SDR). We report total and constrained inertia, the proportion of variance explained by each axis relative to both total and constrained inertia, the adjusted *R* ^2^, and species–environment correlations. Statistical significance of the full model and of each axis was assessed using 998 permutations, with significance determined at *p* < 0.001 (McCune et al. 2002). Multicollinearity among the environmental variables was evaluated using variance inflation factors (VIF) prior to interpreting individual environmental vectors. The MRPP test evaluates the statistical significance of differences between predefined groups by comparing within‐group agreement to that expected by chance, based on distance matrices (McCune et al. 2002). T‐statistics were used for pairwise comparison among groups. T‐values of −10 or lower were interpreted as indicating ecologically significant heterogeneity in species composition (McCune et al. 2002). The agreement statistic (A) is a measure of within‐group homogeneity ranging from 0 to 1. Values ≥ 0.1 indicate clear separation and heterogeneity in species composition between groups, whereas values approaching 1 indicate complete separation (McCune et al. 2002; Mielke and Berry 2001).

### 2.4 Zeta‐Diversity and Species Turnover

To quantify species turnover among vegetation–climate zones, the zetadiv package in R (v4.3.2) was used to analyze zeta diversity (Hui and McGeoch 2014; McGeoch et al. 2019). Multi‐site species turnover and shared‐species patterns were interpreted from zeta‐diversity decline curves diversity (Hui and McGeoch 2014; McGeoch et al. 2019). After deriving zeta‐diversity declines for each vegetation–climate zone, root mean square error (RMSE) was calculated to quantify the similarity among curve shapes.

RMSE measures the difference between two curve models and is widely used to evaluate ecological or climate models and compare spatiotemporal patterns (Willmott and Matsuura 2005). Lower RMSE values indicate greater similarity in curve shape, reflecting similar species turnover structure and community composition patterns between zone. In this study, EZ were compared with each core vegetation–climate zone.

Zeta‐diversity was calculated up to a zeta order of 50 for each vegetation–climate zone diversity (McGeoch et al. 2019). Limiting the analyses to ≤ 50 prevents instability at higher orders, where zeta diversity approaches zero, and enables reliable interpretation of species turnover structure (Hui and McGeoch 2014; McGeoch et al. 2019). Statistical significance of RMSE values was assessed using 2000 bootstrap iterations. The RMSE calculation method is presented below.

(4) $$\text{RMSE}=\sqrt{\frac{1}{50}\sum_{i=1}^{50}{\left(x_{i}-y_{i}\right)}^{2}}$$

where *x*_*i*_ and *yi* represent the zeta‐diversity values of the two curves at the same zeta order.

The zeta‐ratio was analyzed to evaluate species persistence and regional similarity diversity (Hui and McGeoch 2014; McGeoch et al. 2019). The zeta‐ratio represents the probability that a species appearing in the (*n*–1)th survey plot will also occur in the nth survey plot and is calculated using the following equation (Hui and McGeoch 2014; McGeoch et al. 2019).

(5) $$\zeta_{\text{ratio}}\left(i\right)=\frac{\zeta_{i+1}}{\zeta_{i}}$$

ζi: the ith zeta‐diversity (the mean number of shared species among i survey plots). ζi + 1: the (i + 1)th zeta diversity.

To infer ecological assembly processes in EZ and vegetation–climate zones, power‐law and exponential models were compared. The exponential model interprets species turnover as a stochastic process based on neutral theory, whereas the power‐law model interprets it as a deterministic process driven by niche‐ and environment‐based filtering (Hui and McGeoch 2014; McGeoch et al. 2019). Model fit was assessed using the Akaike information criterion (AIC). AIC evaluates the fit of statistical models while accounting for predictive performance and model complexity, thereby reducing the risk of overfitting. AIC values may be positive or negative, and smaller values indicate a better‐fitting model (Akaike 1974).

## 3 Results

### 3.1 Spatial Classification of Ecotones Based on Entropy Thresholds

**FIGURE 2:**
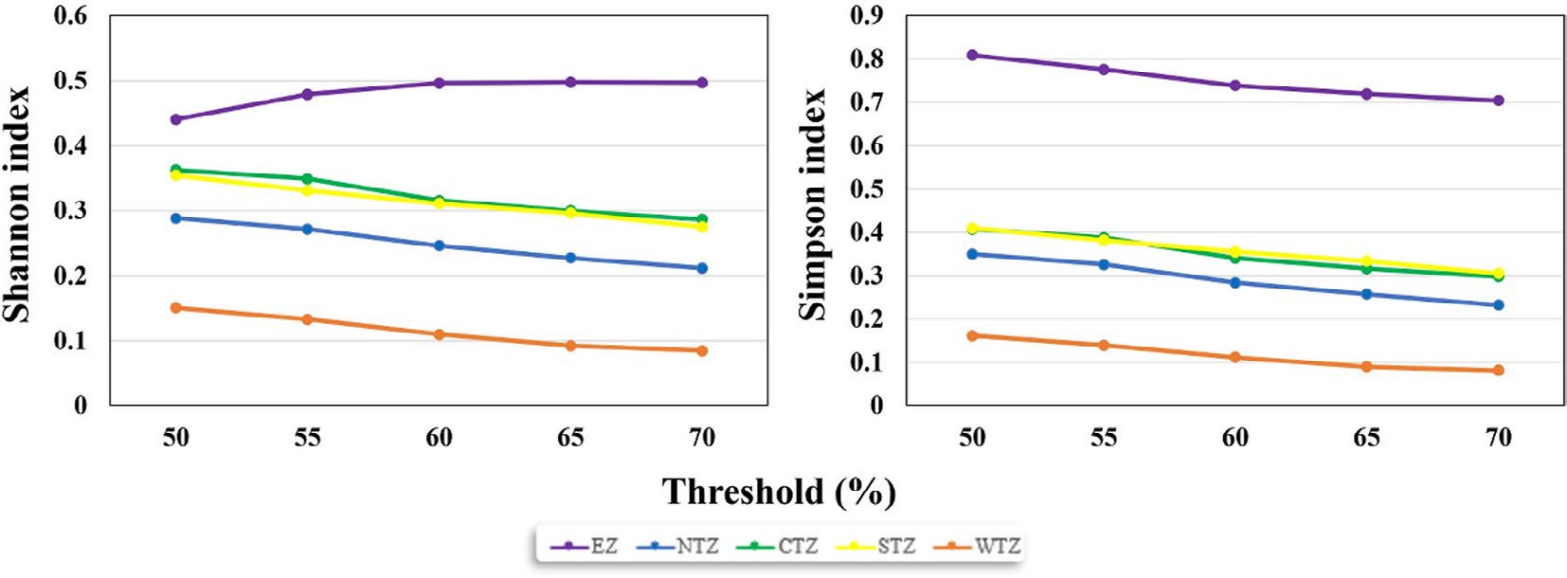
Spatial variation of Shannon entropy across vegetation–climate zones.

The Shannon and Simpson entropy indices were used to quantify the heterogeneity of vegetation–climate zone compositions within the 11 × 13 km grid (Figure 2).

Cells classified as EZ consistently showed the highest Shannon and Simpson index values across all thresholds. In particular, Shannon entropy in EZ increased from 0.441 to 0.497 as the threshold increased from 50% to 70%. In contrast, the Simpson index decreased slightly from 0.809 to 0.704, reflecting a slight reduction in homogeneity in vegetation–climate zone composition while certain vegetation–climate types became increasingly dominant.

The core vegetation–climate zones (CTZ, NTZ, STZ, WTZ) showed lower entropy values than EZ, and the differences increased as the threshold increased. At a 70% threshold, the Shannon index for EZ (0.497) was approximately 1.5–6 times higher than those of CTZ (0.286), NTZ (0.212), STZ (0.275), and WTZ (0.085).

At the 70% threshold, EZ accounted for approximately 29% of the total analyzed area and were primarily distributed in the central and northern regions of South Korea. The entropy separation between EZ and core zones remained stable across thresholds (ΔShannon = 0.468–0.510; Table S1) and across grid sizes (ΔShannon = 0.446–0.462; Table S2), indicating that EZ identification was robust to both threshold choice and spatial resolution. Validation against independent species data showed that EZ occupied intermediate positions in endemic, endangered, and total species richness relative to neighboring vegetation–climate zones (Kruskal–Wallis, all *p* < 0.001; Table S3). Alien species richness was the sole exception, peaking in EZ (see Discussion).

### 3.2 Comparison of Environmental and Ecological Factors

Table 1 presents the comparison of environmental and ecological variables among vegetation–climate zones. EZ showed intermediate values for all variables (Table 1). Statistically significant differences were observed for eight variables, indicating clear environmental and ecological differentiation among vegetation–climate zones (*p* < 0.01).

**TABLE 1:** Results of one‐way ANOVA comparing environmental and ecological indices among vegetation–climate zones.

Index	EZ	NTZ	CTZ	STZ	WTZ
Temperature (°C)**	11.21 ± 0.07^c^	8.44 ± 0.08^e^	10.82 ± 0.05^d^	11.97 ± 0.04^b^	13.45 ± 0.04^a^
Precipitation (mm)**	1263.21 ± 7.21^c^	1316.76 ± 7.76^b^	1262.61 ± 4.45^c^	1216.16 ± 6.24^d^	1376.25 ± 10.70^a^
Slope (°)**	14.90 ± 0.35^b^	21.59 ± 0.29^a^	12.96 ± 0.36^c^	5.35 ± 0.37^e^	7.17 ± 0.36^d^
Altitude (m)**	270.41 ± 10.64^b^	688.69 ± 16.43^a^	203.70 ± 8.13^c^	42.70 ± 4.37^d^	73.38 ± 5.52^d^
Ecosystem service index					
HQ**	0.696 ± 0.009^b^	0.841 ± 0.005^a^	0.630 ± 0.013^c^	0.430 ± 0.017^d^	0.584 ± 0.011^c^
WY (Mm^3^/grid/year)**	352.98 ± 5.85^c^	444.49 ± 7.17a^a^	340.44 ± 5.84^c^	285.02 ± 10.77^d^	391.87 ± 9.41^b^
SDR (ton/grid/year)**	45.74 ± 1.43^b^	68.34 ± 2.25^a^	35.60 ± 1.33^c^	12.09 ± 0.81^d^	33.65 ± 1.44^c^
Bird diversity **	19.77 ± 0.27^c^	16.05 ± 0.26^d^	20.67 ± 0.22^bc^	25.12 ± 0.79^a^	22.21 ± 0.34^b^

*Note:* Different letters indicate significant differences among vegetation–climate zones based on Tukey's post hoc test (*p* < 0.05). Values are presented as mean ± SE (**p < 0.01).

Temperature and altitude were intermediate between NTZ and WTZ, while SDR was relatively high. HQ in EZ (0.696) was second only to NTZ (0.841).

### 3.3 Species Composition Analysis

**FIGURE 3:**
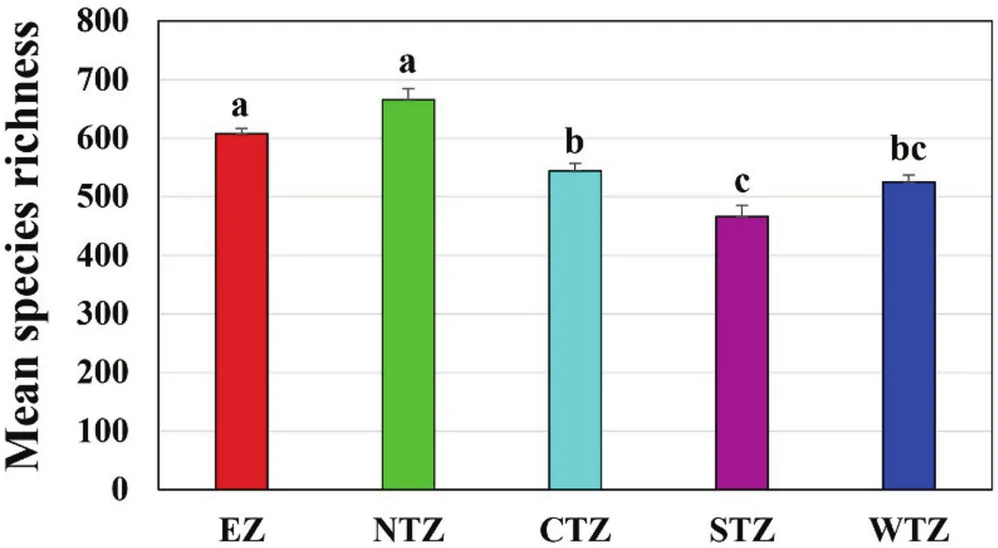
Comparison of mean species richness across five vegetation–climate zones (F = 20.443, *p* < 0.001). Different letters above the bars indicate significant differences based on Tukey's post hoc (*p* < 0.05). Error bars represent standard errors (± SE).

To investigate differences in biodiversity among vegetation–climate zones, grid‐based mean species richness analysis (Figure 3) and CCA were performed. Mean species richness in EZ was 607.8 ± 9.6, the second highest among all groups. The highest species richness was observed in NTZ (665.7 ± 18.5), and followed by CTZ (544.1 ± 13.5), WTZ (524.8 ± 12.8), and STZ (466.9 ± 18.9).

Statistical analysis showed no significant difference between EZ and NTZ (*p* = 0.069), whereas EZ exhibited significantly higher species richness than CTZ and STZ (*p* < 0.01).

**FIGURE 4:**
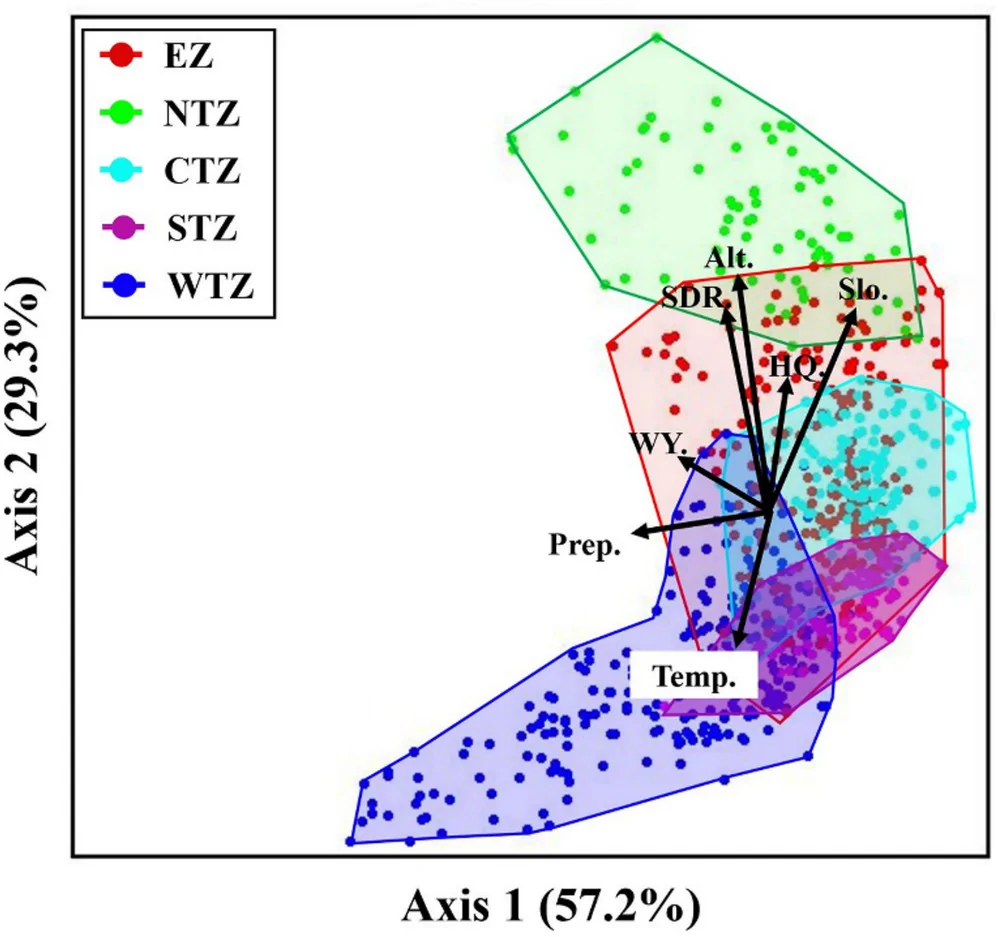
Canonical correspondence analysis (CCA) ordination diagram of species composition across five vegetation–climate zones. The first two canonical axes accounted for 86.5% of the constrained variance (CCA1 = 57.2%, CCA2 = 29.3%). Permutation tests (998 runs) confirmed that the two axes were highly significant (*p* = 0.001). Black arrows (cut off = 0.2) indicate significant environmental variables correlated with species composition: Temperature (Temp.), precipitation (Prep.), water yield (WY), habitat quality (HQ), sediment delivery ratio (SDR), altitude (Alt.), and slope (Slo.).

The total inertia of the species data was 7.60, of which the eight environmental variables explained 0.49 (constrained inertia; 6.4% of the total; adjusted R^2^ = 0.055). Of this constrained variation, the first two axes accounted for 86.5% (CCA1 = 57.2%, CCA2 = 29.3%), corresponding to 4.2% of the total inertia (Figure 4; Table S5). The relatively low proportion of total inertia explained is expected for a species matrix of this size (4712 species) and does not indicate a weak model, as the species–environment Pearson correlations were high (0.966 for CCA1 and 0.887 for CCA2) and the full model and first axis were statistically significant (998 permutations, *p* = 0.001). Multicollinearity among the environmental variables was acceptable (all VIF < 10; maximum elevation = 7.10; Table S6), although elevation and slope were interpreted with caution owing to their moderate intercorrelation (*r* = 0.84). The four core vegetation–climate zones were clearly differentiated along CCA1 and CCA2, while EZ were located near the center of the CCA plane. In a contour overlay, species composition distributions of all vegetation–climate zones overlapped with EZ.

Analysis of correlations among environmental variables showed that temperature was strongly correlated with precipitation, slope, altitude, SDR, HQ, and WY (cut‐off = 0.2).

Overall, the analysis shows that differences in species composition among vegetation–climate zones are strongly associated with environmental factors and ecosystem service indices. In particular, EZ were visually identified as ecological transition zones characterized by complex interactions among these factors.

MRPP analysis was performed to test statistical differences in species composition among vegetation–climate zones (Table 2). The results showed significant differences in species composition between groups (*p* < 0.001), indicating that each vegetation–climate zone retained a distinct species composition structure.

**TABLE 2:** Results of multi‐response permutation procedure (MRPP) comparing species composition among vegetation–climate zones.

Compared groups	T	A	*p*
EZ vs. NTZ	−96.827	0.1626	All pairwise comparisons < 0.001
EZ vs. CTZ	−27.714	0.0317	
EZ vs. STZ	−56.526	0.0759	
EZ vs. WTZ	−146.684	0.1264	
WTZ vs. STZ	−57.197	0.0643	
WTZ vs. CTZ	−147.401	0.1648	
WTZ vs. NTZ	−162.703	0.2353	
STZ vs. CTZ	−52.648	0.0949	
STZ vs. NTZ	−96.916	0.3258	
CTZ vs. NTZ	−93.009	0.2308	

*Note:* T statistics indicate greater heterogeneity between groups, and higher A statistics represent stronger within‐group agreement. Significant differences among groups were confirmed at *p* < 0.001.

The key contrast relevant to our hypotheses was that EZ showed the lowest A‐value with CTZ (A = 0.0317), the greatest compositional overlap among all pairwise comparisons, whereas EZ–NTZ and EZ–WTZ showed higher A‐values, indicating clearer compositional separation (Table 2). Although the EZ–CTZ value falls below the A ≥ 0.1 threshold for strong differentiation (McCune et al. 2002), the comparison remained statistically significant (*p* < 0.001). Full pairwise T‐ and A‐values are provided in Table 2.

### 3.4 Zeta‐Diversity and Species Turnover

**FIGURE 5:**
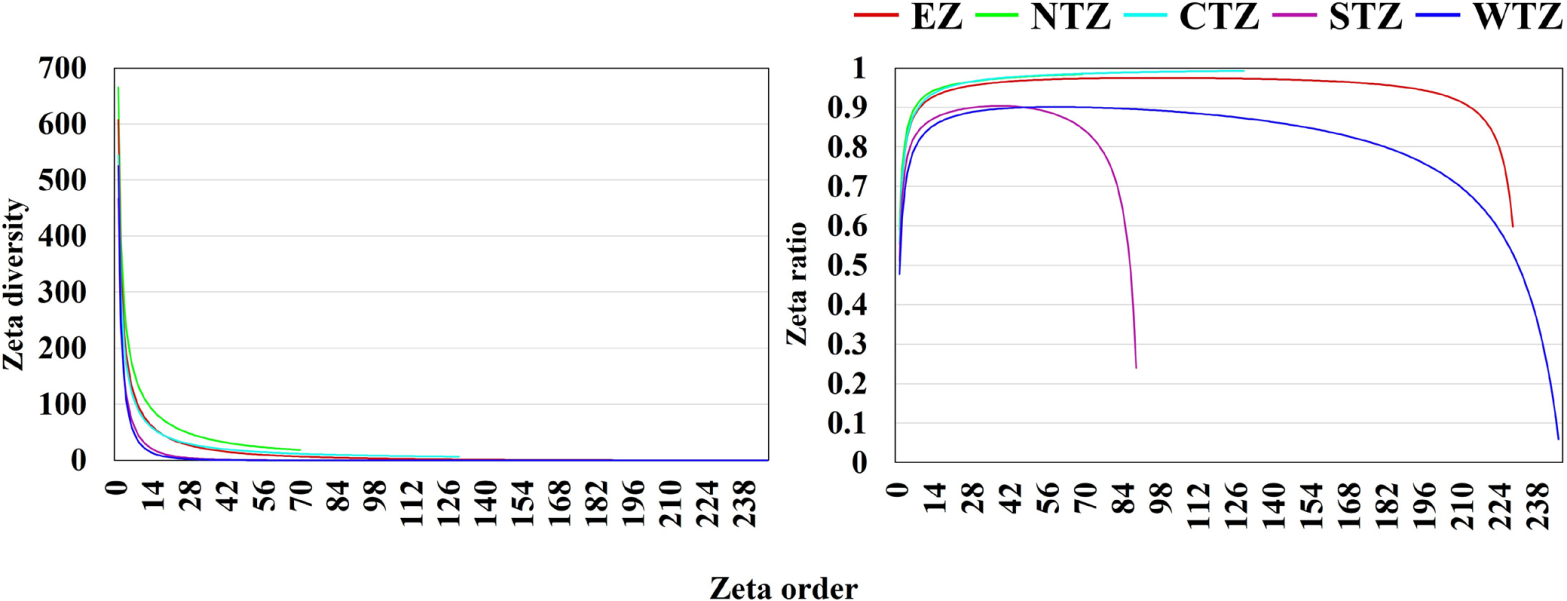
Zeta‐diversity decline (left) and zeta ratio (right) across five vegetation–climate zones (EZ, NTZ, CTZ, STZ, WTZ). EZ‐CTZ showed highly similar decline patterns, with the lowest RMSE value (11.96), indicating compositional similarity. In contrast, STZ, WTZ, and EZ exhibited unimodal zeta‐ratio curves, reflecting localized compositional turnover followed by partial stabilization.

To quantify differences in shared species structure among vegetation–climate zones, zeta‐diversity was analyzed (Figure 5).

Across all vegetation–climate zones, increasing zeta order was accompanied with an exponential decrease in zeta diversity, reflecting typical species turnover patterns in which the number of shared species declines sharply as the order increases.

The decline curve for EZ was intermediate relative to those of NTZ, STZ, and WTZ, indicating species turnover characteristics that were neither highly dynamic (high turnover) nor highly stable (low turnover).

NTZ showed the shallowest decline among all groups. The zeta ratio tended to converge toward 1 as zeta order increased in most zones, whereas STZ, WTZ, and EZ showed unimodal patterns.

RMSE analysis of the zeta‐diversity decline curves (Table 3) confirmed that EZ and CTZ had the most similar turnover patterns (lowest RMSE among EZ comparisons), consistent with their overlapping decline curves. Detailed RMSE values for all comparisons are provided in Table 3. In all comparisons, bootstrap‐based *p*‐values were ≥ 0.5, indicating no statistical significance. Nevertheless, RMSE was interpreted as a supporting metric for evaluating relative similarity among curves rather than as a formal statistical test.

**TABLE 3:** Root mean square error (RMSE) values comparing zeta‐diversity decline curves among vegetation–climate zones.

Compared groups	RMSE	95% CI (min–max)	*p*
EZ vs. NTZ	28.18	24.66–31.69	0.532
EZ vs. CTZ	11.96	5.29–18.35	0.563
EZ vs. STZ	41.36	31.13–51.94	0.546
EZ vs. WTZ	42.09	34.82–48.93	0.517
WTZ vs. STZ	9.97	4.76–15.42	0.563
WTZ vs. CTZ	36.32	31.75–40.88	0.513
WTZ vs. NTZ	69.96	59.28–80.24	0.522
STZ vs. CTZ	33.70	29.29–38.30	0.530
STZ vs. NTZ	68.91	55.58–82.83	0.544
CTZ vs. NTZ	35.83	26.46–45.22	0.545

To ascertain the ecological mechanisms underlying zeta‐diversity declines, AIC values of exponential and power‐law models were compared (Table 4).

**TABLE 4:** AIC values of exponential (Exp.) and power‐law (PL) models fitted to zeta‐diversity decline curves for each vegetation–climate zone.

Index	Exp.	Pl.
EZ	31.0623*	373.8543
NTZ	−87.9591	−286.7744*
CTZ	−91.7867	−720.51775*
STZ	88.8223*	232.5401
WTZ	1026.1650*	1359.9460

*Note:* Asterisks (*) indicate the model supported for each vegetation–climate zone (i.e., the lower AIC value).

In EZ, the exponential model showed a lower AIC value (31.06) than the power‐law model (373.85), indicating the dominance of stochastic processes. This indicates that species turnover in EZ is influenced less by deterministic environmental factors and more by random spatial distribution and intermediate disturbance.

However, this stochastic turnover does not indicate ecological instability. Instead, it indicates that EZ maintain relative stability while sharing species composition with multiple climate zones, providing quantitative evidence for their role as ecological buffer zones.

In contrast, in CTZ, the power‐law model showed a lower AIC value (−720.52) than the exponential model (−91.79), indicating a stronger influence of deterministic assembly mechanisms. This indicates that CTZ is more directly affected by environmental gradients (particularly temperature and precipitation) and that species composition is structured by environmental filtering.

Although EZ and CTZ showed the highest similarity in species turnover in the zeta‐diversity decline and RMSE analyses, different ecological assembly models were supported for each zone. This indicates that these two zones form a transitional complex where, despite spatial adjacency, different ecological processes operate.

The coexistence of these mechanisms may drive long‐term dynamic turnover in EZ and contribute to their sensitivity to biodiversity changes under climate change. For NTZ, the power‐law model showed a lower AIC value (−286.77) than the exponential model (−87.96), indicating the dominance of deterministic assembly mechanisms. This reflects the strong climatic and topographic constraints of this high‐latitude, high‐altitude environment, where species composition is structured by environmental filtering. As such, NTZ contains species adapted to extreme conditions and is ecogeographically important as a potential refugium under climate change.

Meanwhile, STZ and WTZ showed positive AIC values for both models, but the exponential model (AIC = 88.82 and 1026.17) showed a better fit, indicating the relative dominance of stochastic processes. This indicates ongoing species dispersal under relatively stable climatic conditions and facilitated exchange between regions. In other words, these zones function as open ecosystems with gradual spatial turnover due to weaker environmental constrictions.

In summary, our results show that EZ follow a stochastic turnover process while also being influenced by deterministic processes from the adjacent CTZ. Therefore, EZ function as ecological junctions where two mechanisms for ecological change coexist and interact. This complexity contributes to the high heterogeneity and ecological sensitivity of EZ.

## 4 Discussion

### 4.1 Environmental Characteristics and Buffering Role of Ecotone Zones

The consistently higher Shannon and Simpson entropy values in EZ relative to all core zones (Section 3.1) indicate that EZ function as transition zones characterized by a high diversity of vegetation types, consistent with their landscape‐scale definition as ecotone‐probable grids (Section 2.1). Across environmental and ecosystem‐service variables, EZ exhibited intermediate values (Section 3.2), and this transitional positioning suggests that EZ do not simply average the conditions of adjacent zones but instead integrate distinct thermal, topographic, and hydrological gradients into a single spatial unit, functioning as structural buffers (Jiang et al. 2012). This structural diversity has been linked to temperate forest productivity and improved ecosystem functioning (Park, Park, et al. 2019).

Consistent with this interpretation, the relatively high SDR observed in EZ (Section 3.2) may reflect active soil material circulation associated with their transitional topographic position, while the elevated HQ (0.696, second only to NTZ) suggests generally favorable habitat conditions within these zones.

The coexistence of multiple vegetation–climate zones within a limited area indicates that EZ increase environmental heterogeneity and may enhance resilience to climate change or disturbance through various feedback mechanisms (Ries et al. 2004; Hufkens et al. 2009). Such transition zones are generally understood to improve ecosystem stability by dispersing ecological stress spatially and maintaining biological connectivity between neighboring habitats (Kark 2012). In the monsoon climate and complex topography of South Korea (Moon et al. 2017), this buffering function may be particularly relevant: Mid‐latitude EZ at the boundaries of deciduous broadleaf and mixed coniferous–broadleaf forests can function as corridors for species migration and help maintain biodiversity under rapid changes in temperature or moisture (Lee et al. 2025).

Maintaining the structural and environmental integrity of EZ may therefore contribute to the climate‐change resilience and adaptability of forest ecosystems.

### 4.2 Species Richness and Compositional Overlap With Adjacent Zones

The near‐parity in species richness between EZ and NTZ—two zones with markedly different environmental templates—points to distinct underlying mechanisms converging on a similar richness outcome: environmental filtering in the climatically extreme NTZ versus species‐pool mixing in the environmentally intermediate EZ. This pattern is consistent with the mid‐domain effect, in which biodiversity peaks in the intermediate portions of environmental gradients as a result of overlapping species ranges (Colwell and Lees 2000). EZ represent areas where climatic and topographic conditions overlap, creating habitat conditions that facilitate the coexistence of diverse plant species, as reported for mountainous and temperate forest transition zones where species composition and structural diversity are maximized in mid‐altitude or transitional environments (Rahbek 1995; Grytnes 2003).

However, elevated richness in transitional zones does not intrinsically indicate ecological stability. EZ contain diverse habitat patches and maintain high biodiversity but remain in a state of dynamic equilibrium that habitat fragmentation or boundary expansion from human activity could easily disrupt (Jetz and Fine 2012; Haddad et al. 2015). Consistent with this, our independent validation showed that alien species richness peaked in EZ rather than in any core zone (Table S2), indicating that the same edge conditions that elevate native and endemic richness also render EZ susceptible to invasion by disturbance‐tolerant and alien species. EZ therefore combine high conservation value with heightened invasion risk, and species composition—particularly the influx of ecologically disruptive alien species—should be prioritized for biodiversity monitoring.

By contrast, the southern temperate mixed evergreen–deciduous broadleaf forest zone (WTZ) showed relatively low species abundance despite occupying the largest area (32.9%) of the analyzed grid, contrary to the species–area hypothesis, which predicts that richness typically increases with area (Connor and McCoy 1979; McGuinness 1984). Climatically stable and homogeneous environments have been reported to maintain lower richness (Currie 1991); as WTZ has a relatively stable climate and simple topographic structure, it provides fewer ecological niches and exhibits high homogeneity in species composition.

These patterns were also evident in the CCA, where EZ were located near the center of the environmental vectors and reflected factors from several neighboring zones simultaneously, indicating that species composition in EZ is strongly influenced by environmental gradients. Although this may appear to contrast with the stochastic turnover observed in the zeta‐diversity analyses, the difference likely arises from scale effects: Stochastic assembly may dominate at broader spatial scales among zones, whereas environmental filtering remains influential at intra‐zonal scales (Leibold et al. 2004; Chase and Myers 2011). This filtering signal is reflected in the strong correlation of temperature with precipitation, slope, altitude, SDR, HQ, and WY, pointing to temperature as a key factor representing topographic stability and ecological condition across zones, while the combination of intermediate altitude and SDR with relatively high HQ in EZ indicates that these areas are both physically and ecologically heterogeneous, consistent with their central position in the CCA ordination space.

The compositional continuity between EZ and CTZ, contrasted with the sharper separation from NTZ and WTZ (Section 3.3), suggests that EZ assembly is shaped less by uniform mixing of all neighboring pools and more by asymmetric dispersal from the climatically and topographically closer CTZ, consistent with a source–sink framework in which propagule pressure from the nearest, most similar zone disproportionately structures the transitional community (Kark 2012). The elevated richness in EZ (607.8 ± 9.6) may thus reflect the mixing of species pools from adjacent zones rather than the presence of unique ecotone‐specialist species, consistent with the species‐mixing hypothesis (Kark 2012) and the intermediate position of EZ in the CCA ordination space. Under future disturbance or intensified climate change, turnover between EZ and CTZ may either stabilize or accelerate (Allen and Breshears 1998), and increased sensitivity to disturbance may in the long term accelerate species reassembly or ecotone shifts (Gosz 1993). Overall, EZ function as mediation zones where continuity and heterogeneity of species composition coexist, so that their compositional similarity with neighboring zones can be read both as an indicator of ecosystem stability and as an early signal of ecological change—reinforcing the rationale for prioritizing EZ in protected‐area management and biodiversity monitoring.

### 4.3 Species Turnover and Community Assembly Processes

In the zeta‐diversity analyses, all vegetation–climate zones showed the typical exponential decrease in shared species with increasing zeta order. In EZ, however, rapid turnover in localized composition at low zeta orders (ζ_1_–ζ_10_) gave way to a unimodal pattern at higher orders (≥ζ_24_), indicating that EZ initially contain mixtures of species from multiple zones but become dominated by specific assemblages as spatial scale increases—functioning both as compositional buffers and as habitats characterized by turnover and partial stability (Gosz 1993; Risser 1995). Consistent with this, the intermediate zeta‐diversity decline of EZ relative to NTZ, STZ, and WTZ (Section 3.4) indicates a transitional (ecotonal) structure that partially shares composition with surrounding zones while retaining a degree of compositional independence. The unimodal zeta‐ratio patterns shared by EZ, STZ, and WTZ further suggest partial compositional stability at intermediate zeta orders, consistent with the “ecotone resilience” concept of Jiang et al. (2012); this contrasts with the expectation of highly dynamic boundaries (D'Odorico et al. 2013) and aligns more closely with the “static ecotone” hypothesis of Tölgyesi et al. (2016) and Peringer and Rosenthal (2011), in which dispersal limitation and stochastic processes can maintain stable ecotone positions despite environmental change.

Although EZ and CTZ showed similar zeta‐diversity decline curves, their zeta‐ratio patterns differed, indicating that—despite comparable turnover rates—the two zones differ in species persistence and habitat selectivity: EZ exhibited dynamically balanced structures influenced by complex environmental conditions, whereas CTZ were shaped more strongly by deterministic environmental gradients, as supported by the AIC‐based model comparisons (Leibold et al. 2004; Chase and Myers 2011). The dominance of the exponential model in EZ suggests that stochastic processes—ecological drift, dispersal limitation, and random colonization—play a primary role in community assembly within these transition zones, consistent with metacommunity theory (Leibold et al. 2004) and the finding that stochastic processes become increasingly important under high environmental heterogeneity or intermediate disturbance (Chase and Myers 2011).

By contrast, NTZ showed a shallow zeta‐diversity decline and supported a power‐law model, indicating strong structuring by environmental filtering under cold, high‐altitude conditions (Vellend 2010; Kraft et al. 2015). Strong climatic constraints in this northern zone limit species turnover with neighboring zones and lead to specialized plant communities, so that NTZ maintains a heterogeneous species composition and may function as a refugium under climate change (Dobrowski 2011). The STZ, in turn, showed the lowest species abundance and may represent a region where environmental warming and stabilization are simplifying community structure through dominance by certain species.

Finally, rising temperatures have been associated with gradual northward shifts of EZ in temperate forest vegetation (Song et al. 2014). Our results indicate that species composition in South Korean temperate forests is not static but changes spatiotemporally in response to climate and disturbance, consistent with long‐term monitoring studies reporting continuous variation in turnover rates driven by interactions among climate, soil, and topographic factors (Jung et al. 2021; Park and Cheon 2025). EZ in South Korea should therefore be regarded as dynamic habitat boundaries under changing climatic conditions.

### 4.4 Study Limitation

Several limitations should be noted. First, the 11 × 13 km grid resolution may obscure fine‐scale ecotone boundaries, which can range from meters to kilometers (Brownstein et al. 2015). This resolution was adopted from the national survey's standard unit and reflects our landscape‐scale focus on identifying grid cells with a high probability of ecotonal structure rather than delineating individual boundaries; a grid‐size sensitivity analysis confirmed that EZ classification is robust across resolutions (Table S2). Second, the Nationwide Survey data may carry spatial sampling biases toward accessible areas, which could underrepresent species richness in remote high‐altitude NTZ cells. Third, the vegetation‐climate zone boundaries used as input data carry inherent classification uncertainty (Rocchini et al. 2013; Feilhauer et al. 2021). Fourth, the climate data (WorldClim 1970–2000 normals) and the vegetation survey (2006–2023) differ in their reference periods. We used 30‐year climatic normals to represent the long‐term spatial climatic gradient to which vegetation–climate zones respond; because the south–north spatial gradient in annual mean temperature across South Korea (approximately 6.4°C, from 16.6°C in the far south to 10.2°C in the far north; Park, Kim, et al. 2019) is roughly an order of magnitude larger than the warming over the intervening period (approximately 0.3°C, at a non‐urban station rate of 0.11°C decade^−1^; Kim et al. 2016), the choice of reference period does not materially alter the spatial gradient structure. Nevertheless, our climate–vegetation relationships should be interpreted as correlative associations along a long‐term spatial gradient rather than as temporally matched climate effects. Finally, our analysis is cross‐sectional and cannot capture temporal shifts in EZ position in response to climate change, which is a critical dimension for conservation planning (Virtanen et al. 2010).

## 5 Conclusion

In this study, the spatial distribution and ecological characteristics of EZ were quantitatively assessed across temperate forest vegetation–climate zones in South Korea. EZ exhibited intermediate environmental conditions and transitional species composition relative to adjacent regions, together with high entropy and intermediate levels of species turnover—patterns consistent with a transitional, buffering character. These patterns suggest that EZ may contribute to biodiversity maintenance, although their functional roles and responses to climate change were not directly tested here and warrant dedicated investigation.

In the future, more precise identification of indicator species and ecotone delineation could be achieved by integrating population counts or cover‐based quantitative data.

Long‐term ecological monitoring of EZ is also important for the early detection of habitat changes that are particularly sensitive to climate change and could serve a core indicator for proactive conservation and the development of national‐level management strategies.

As a central biodiversity conservation framework, the aim of KMGBF was to protect or restore at least 30% of land and sea areas by 2030 (Target 3). Achieving this international goal requires both quantitative expansion of protected areas and scientifically credible studies of diverse habitat environments. The EZ identified in this study occur at the intersection of climatic and vegetation gradients and are characterized by high species diversity and turnover. These patterns suggest that EZs may merit consideration as candidate areas in protected‐area planning, though translating these spatial patterns into specific management decisions will require further assessment of their conservation outcomes.

## Author Contributions

**Byeong‐Joo Park:** conceptualization (lead), data curation (lead), formal analysis (lead), investigation (lead), methodology (lead), supervision (supporting), visualization (lead), writing – original draft (lead), writing – review and editing (supporting). **Eun‐Seo Lee:** funding acquisition (supporting), investigation (supporting), writing – review and editing (supporting). **Kwangil Cheon:** funding acquisition (lead), investigation (supporting), project administration (lead), writing – review and editing (lead).

## Funding

This research was conducted as part of the National Institute of Ecology's “Study on the Thematic and Demand Assessment of Ecosystem Services” research project (Project No. NIE‐B‐2026‐03) and the Korea Environment Industry & Technology Institute (KEITI) under the Climate Change R&D Project for the New Climate Regime (RS‐2022‐KE002369).

## Conflicts of Interest

The authors declare no conflicts of interest.

## Supporting information


**Table S1:** Threshold‐sensitivity analysis for ecotone zone (EZ) classification.
**Table S2:** Grid‐size sensitivity analysis for EZ classification.
**Table S3:** Independent ecological validation of EZ classification using species data not used in the entropy‐based delineation.
**Table S4:** InVEST model input data, sources, and parameter settings for the ecosystem‐service indices (HQ, WY, SDR).
**Table S5:** Canonical correspondence analysis (CCA): partitioning of total and constrained inertia. Total inertia in species data: 7.6046. Constrained inertia (species–environment): 0.4884. Adjusted *R* ^2^ = 0.0548. Global permutation *p* = 0.001.
**Table S6:** Variance inflation factors (VIF) for the environmental variables used in CCA.

**Data S1:** ece374306‐sup‐0002‐supinfo.xlsx.

## Data Availability

All the required data are uploaded as Supporting Information.

## References

[ece374306-bib-0001] Akaike, H. 1974. “A New Look at the Statistical Model Identification.” IEEE Transactions on Automatic Control 19: 716–723. 10.1109/TAC.1974.1100705.

[ece374306-bib-0002] Allen, C. D. , and D. D. Breshears . 1998. “Drought‐Induced Shift of a Forest–Woodland Ecotone: Rapid Landscape Response to Climate Variation.” Proceedings of the National Academy of Sciences of the United States of America 95, no. 25: 14839–14842. 10.1073/pnas.95.25.14839. 9843976 PMC24536

[ece374306-bib-0003] Brownstein, G. , C. Johns , A. Fletcher , D. Pritchard , and P. D. Erskine . 2015. “Ecotones as Indicators: Boundary Properties in Wetland‐Woodland Transition Zones.” Community Ecology 16, no. 2: 235–243. 10.1556/168.2015.16.2.11.

[ece374306-bib-0004] Chase, J. M. , and J. A. Myers . 2011. “Disentangling the Importance of Ecological Niches From Stochastic Processes Across Scales.” Philosophical Transactions. Series B, Biological Sciences 366, no. 1576: 2351–2363. 10.1098/rstb.2011.0063. PMC3130433 21768151

[ece374306-bib-0005] Choe, H. , J. H. Thorne , P. R. Huber , D. Lee , and J. F. Quinn . 2018. “Assessing Shortfalls and Complementary Conservation Areas for Plant Biodiversity in South Korea.” PLoS One 13, no. 2: e0190754. 10.1371/journal.pone.0190754. 29474355 PMC5825007

[ece374306-bib-0006] Collinge, S. K. 2009. Ecology of Fragmented Landscapes. Johns Hopkins University Press Baltimore.

[ece374306-bib-0007] Colwell, R. K. , and D. C. Lees . 2000. “The Mid‐Domain Effect: Geometric Constraints on the Geography of Species Richness.” Trends in Ecology & Evolution 15, no. 2: 70–76. 10.1016/S0169-5347(99)01767-X. 10652559

[ece374306-bib-0008] Connor, E. F. , and E. D. McCoy . 1979. “The Statistics and Biology of the Species‐Area Relationship.” American Naturalist 113, no. 6: 791–833. 10.1086/283438.

[ece374306-bib-0009] Convention on Biological Diversity . 2024. “Kunming–Montreal Global Biodiversity Framework.” https://www.cbd.int/gbf.

[ece374306-bib-0010] Currie, D. J. 1991. “Energy and Large‐Scale Patterns of Animal‐ and Plant‐Species Richness.” American Naturalist 137, no. 1: 27–49. 10.1086/285144.

[ece374306-bib-0011] De Klerk, H. M. , N. D. Burgess , and V. Visser . 2018. “Probabilistic Description of Vegetation Ecotones Using Remote Sensing.” Ecological Informatics 46: 125–132. 10.1016/j.ecoinf.2018.06.001.

[ece374306-bib-0012] Dobrowski, S. Z. 2011. “A Climatic Basis for Microrefugia: The Influence of Terrain on Climate.” Global Change Biology 17, no. 2: 1022–1035. 10.1111/j.1365-2486.2010.02263.x.

[ece374306-bib-0013] D'Odorico, P. , Y. He , S. Collins , S. F. J. de Wekker , V. Engel , and J. D. Fuentes . 2013. “Vegetation–Microclimate Feedbacks in Woodland–Grassland Ecotones.” Global Ecology and Biogeography 22, no. 4: 364–379. 10.1111/geb.12000.

[ece374306-bib-0014] Feilhauer, H. , A. Zlinszky , A. Kania , et al. 2021. “Let Your Maps be Fuzzy!—Class Probabilities and Floristic Gradients as Alternatives to Crisp Mapping for Remote Sensing of Vegetation.” Remote Sensing in Ecological and Conservation 7, no. 2: 292–305. 10.1002/rse2.188.

[ece374306-bib-0015] Gosz, J. R. 1993. “Ecotone Hierarchies.” Ecological Applications 3, no. 3: 369–376. 10.2307/1941905. 27759251

[ece374306-bib-0016] Gottfried, M. , H. Pauli , and G. Grabherr . 1998. “Prediction of Vegetation Patterns at the Limits of Plant Life: A New View of the Alpine‐Nival Ecotone.” Arctic and Alpine Research 30, no. 3: 207–221. 10.1080/00040851.1998.12002894.

[ece374306-bib-0017] Grytnes, J. A. 2003. “Species–Richness Patterns of Vascular Plants Along Seven Altitudinal Transects in Norway.” Ecography 26, no. 3: 291–300. 10.1034/j.1600-0587.2003.03358.x.

[ece374306-bib-0018] Haddad, N. M. , L. A. Brudvig , J. Clobert , et al. 2015. “Habitat Fragmentation and Its Lasting Impact on Earth's Ecosystems.” Science Advances 1, no. 2: e1500052. 10.1126/sciadv.1500052. 26601154 PMC4643828

[ece374306-bib-0019] Hufkens, K. , P. Scheunders , and R. Ceulemans . 2009. “Ecotones in Vegetation Ecology: Methodologies and Definitions Revisited.” Ecological Research 24, no. 5: 977–986. 10.1007/s11284-009-0584-7.

[ece374306-bib-0020] Hui, C. , and M. A. McGeoch . 2014. “Zeta Diversity as a Concept and Metric That Unifies Incidence‐Based Biodiversity Patterns.” American Naturalist 184, no. 5: 684–694. 10.1086/678125. 25325751

[ece374306-bib-0021] Jetz, W. , and P. V. A. Fine . 2012. “Global Gradients in Vertebrate Diversity Predicted by Historical Area‐Productivity Dynamics and Contemporary Environment.” PLoS Biology 10, no. 3: e1001292. 10.1371/journal.pbio.1001292. 22479151 PMC3313913

[ece374306-bib-0022] Jiang, J. , D. Gao , and D. L. DeAngelis . 2012. “Towards a Theory of Ecotone Resilience: Coastal Vegetation on a Salinity Gradient.” Theoretical Population Biology 82, no. 1: 29–37. 10.1016/j.tpb.2012.02.007. 22441163

[ece374306-bib-0023] Jost, L. 2006. “Entropy and Diversity.” Oikos 113, no. 2: 363–375. 10.1111/j.2006.0030-1299.14714.x.

[ece374306-bib-0024] Jung, S. , H. S. Sim , J. S. Kim , K. H. Bae , and Y. C. Cho . 2021. “Processes Driving Understory Community Dynamics in Ulleungdo Island Broadleaved Forest, South Korea.” Ecological Research 36, no. 4: 686–700. 10.1111/1440-1703.12231.

[ece374306-bib-0025] Kark, S. 2012. “Ecotones and Ecological Gradients.” In Ecological Systems, edited by R. A. Meyers , 147–160. Springer New York.

[ece374306-bib-0026] Kim, H. S. , Y. S. Chung , P. P. Tans , and M. B. Yoon . 2016. “Climatological Variability of Air Temperature and Precipitation Observed in South Korea for the Last 50 Years.” Air Quality, Atmosphere & Health 9, no. 6: 645–651. 10.1007/s11869-015-0366-z.

[ece374306-bib-0027] Kimmins, J. P. 1997. Forest Ecology: A Foundation for Sustainable Management. Prentice Hall Saddle River, NJ.

[ece374306-bib-0028] Korea National Arboretum . 2020. Forest of Korea (IV) Biogeography of Korea: Flora and Vegetation, 42–79. Sumeungil, Seoul, Republic of Korea.

[ece374306-bib-0029] Körner, C. 2007. “The Use of ‘Altitude’ in Ecological Research.” Trends in Ecology & Evolution 22, no. 11: 569–574. 10.1016/j.tree.2007.09.006. 17988759

[ece374306-bib-0030] Kraft, N. J. B. , P. B. Adler , O. Godoy , E. C. James , S. Fuller , and J. M. Levine . 2015. “Community Assembly, Coexistence and the Environmental Filtering Metaphor.” Functional Ecology 29, no. 5: 592–599. 10.1111/1365-2435.12345.

[ece374306-bib-0031] Lavorel, S. , K. Grigulis , S. McIntyre , et al. 2008. “Assessing Functional Diversity in the Field–Methodology Matters!” Functional Ecology 22, no. 1: 134–147. 10.1111/j.1365-2435.2007.01339.x.

[ece374306-bib-0032] Lee, J. , Y. J. Yoo , N. O. Lim , et al. 2025. “Impact Factor and the Range of Ecological Connectivity Change due to Development.” Environmental Research Communications 7: e041007. 10.1088/2515-7620/adc906.

[ece374306-bib-0033] Leibold, M. A. , M. Holyoak , N. Mouquet , et al. 2004. “The Metacommunity Concept: A Framework for Multi‐Scale Community Ecology.” Ecology Letters 7: 601–613. 10.1111/j.1461-0248.2004.00608.x.

[ece374306-bib-0034] Maestre, F. T. , J. L. Quero , N. J. Gotelli , et al. 2012. “Plant Species Richness and Ecosystem Multifunctionality in Global Drylands.” Science 335: 214–218. 10.1126/science.1215442. 22246775 PMC3558739

[ece374306-bib-0035] Magurran, A. E. 2004. Measuring Biological Diversity. Blackwell Publishing Oxford.

[ece374306-bib-0036] Marfo, T. D. , K. Resjek , and V. Vranova . 2018. “Spatial Variations in Soil Properties Across Ecotones: A Short Review.” Bulletin of Geography Physical Geography Series 14: 71–77. 10.2478/bgeo-2018-0006.

[ece374306-bib-0037] Marimon, B. S. , E. D. S. Lima , T. G. Duarte , L. C. Chieregatto , and J. A. Ratter . 2006. “Observations on the Vegetation of Northeastern Mato Grosso, Brazil. IV. An Analysis of the Cerrado–Amazonian Forest Ecotone.” Edinburgh Journal of Botany 63, no. 2–3: 323–341. 10.1017/S0960428606000576.

[ece374306-bib-0038] McCune, B. , J. B. Grace , and D. L. Urban . 2002. Analysis of Ecological Communities. MjM Software Design Gleneden Beach, OR.

[ece374306-bib-0039] McGeoch, M. A. , G. Latombe , N. R. Andrew , et al. 2019. “Measuring Continuous Compositional Change Using Decline and Decay in Zeta Diversity.” Ecology 100, no. 11: e02832. 10.1002/ecy.2832. 31323117

[ece374306-bib-0040] McGuinness, K. A. 1984. “Species–Area Curves.” Biological Reviews 59, no. 3: 423–440. 10.1111/j.1469-185X.1984.tb00711.x.

[ece374306-bib-0041] Mielke, P. W. , and K. J. Berry . 2001. Permutation Methods: A Distance Function Approach. Springer, New York. 10.1007/978-1-4757-3449-2.

[ece374306-bib-0042] Ministry of Environment . 2023. The Fifth National Biodiversity Strategy (2024–2028). Ministry of Environment Sejong, Republic of Korea.

[ece374306-bib-0043] Moon, J. , W. K. Lee , C. Song , et al. 2017. “An Introduction to Mid‐Latitude Ecotone: Sustainability and Environmental Challenges.” Siberian Journal of Forest Science 6: 41–51. 10.15372/SJFS20170603.

[ece374306-bib-0044] National Institute of Ecology . 2019. Manual of the 5th National Survey of Natural Environment, 11–50. National Institute of Ecology, Seocheon, Republic of Korea.

[ece374306-bib-0045] National Institute of Ecology . 2022. The Manual of Assessment Map of Ecosystem Service, 54–61. Design Crepas, Seoul, Republic of Korea.

[ece374306-bib-0046] Odum, E. P. 1971. Fundamentals of Ecology. W.B. Saunders.

[ece374306-bib-0047] Oliver, C. D. , and B. C. Larson . 1990. Forest Stand Dynamics. McGraw‐Hill.

[ece374306-bib-0049] Park, B. J. , and K. Cheon . 2025. “Species Composition and Ecological Niche Overlap of Alien and Endemic Plants in South Korea: Insights From the National Ecosystem Survey.” Forests 16: 1485. 10.3390/f16091485.

[ece374306-bib-0050] Park, B. J. , S. K. Han , S. H. Park , K. Cheon , S. H. Che , and T. I. Heo . 2024. “Correlation Between Vegetation Structure and Environmental Factors of *Dracocephalum argunense* Habitats in South Korea.” Journal of Korean Society of Forest Science 113, no. 3: 271–281. 10.14578/jkfs.2024.113.3.271.

[ece374306-bib-0051] Park, J. , H. S. Kim , H. K. Jo , and I. B. Jung . 2019. “The Influence of Tree Structural and Species Diversity on Temperate Forest Productivity and Stability in Korea.” Forests 10, no. 12: 1113. 10.3390/f10121113.

[ece374306-bib-0052] Park, S. , H. Park , J. Im , et al. 2019. “Delineation of High Resolution Climate Regions Over the Korean Peninsula Using Machine Learning Approaches.” PLoS One 14: e0223362. 10.1371/journal.pone.0223362. 31600268 PMC6786637

[ece374306-bib-0053] Paz‐Kagan, T. , T. Caras , I. Herrmann , M. Shachak , and A. Karnieli . 2017. “Multiscale Mapping of Species Diversity Under Changed Land Use Using Imaging Spectroscopy.” Ecological Applications 27, no. 5: 1466–1484. 10.1002/eap.1540. 28370671

[ece374306-bib-0054] Peringer, A. , and G. Rosenthal . 2011. “Establishment Patterns in a Secondary Tree Line Ecotone.” Ecological Modelling 222, no. 17: 3120–3131. 10.1016/j.ecolmodel.2011.05.025.

[ece374306-bib-0055] Rahbek, C. 1995. “The Elevational Gradient of Species Richness: A Uniform Pattern?” Ecography 18, no. 2: 200–205.

[ece374306-bib-0056] Ricotta, C. 2005. “Biodiversity Concepts.” Acta Biotheoretica 53: 29–38. 10.1007/s10441-005-2539-5. 15906141

[ece374306-bib-0057] Ries, L. , R. J. Fletcher Jr. , J. Battin , and T. D. Sisk . 2004. “Ecological Responses to Habitat Edges: Mechanisms, Models, and Variability Explained.” Annual Review of Ecology, Evolution, and Systematics 35: 491–522. 10.1146/annurev.ecolsys.35.112202.130148.

[ece374306-bib-0058] Risser, P. G. 1995. “The Status of the Science Examining Ecotones: A Dynamic Aspect of Landscape Is the Area of Steep Gradients Between More Homogeneous Vegetation Associations.” Bioscience 45, no. 5: 318–325. 10.2307/1312492.

[ece374306-bib-0059] Rocchini, D. , G. M. Foody , H. Nagendra , et al. 2013. “Uncertainty in Ecosystem Mapping by Remote Sensing.” Computers & Geosciences 50: 128–135. 10.1016/j.cageo.2012.05.022.

[ece374306-bib-0060] Rocchini, D. , and C. Ricotta . 2007. “Are Landscapes as Crisp as We May Think?” Ecological Modelling 204: 535–539. 10.1016/j.ecolmodel.2006.12.028.

[ece374306-bib-0061] Sekercioglu, C. H. 2006. “Increasing Awareness of Avian Ecological Function.” Trends in Ecology & Evolution 21: 464–471. 10.1016/j.tree.2006.05.007. 16762448

[ece374306-bib-0062] Shannon, C. E. 1948. “A Mathematical Theory of Communication.” Bell System Technical Journal 27, no. 3: 379–423. 10.1002/j.1538-7305.1948.tb01338.x.

[ece374306-bib-0063] Simpson, E. H. 1949. “Measurement of Diversity.” Nature 163: 688. 10.1038/163688a0.

[ece374306-bib-0064] Song, K. , T. S. Kohyama , and L. J. Da . 2014. “Transition Patterns Across an Evergreen–Deciduous Broad‐Leaved Forest Ecotone: The Effect of Topographies.” Journal of Vegetation Science 25, no. 5: 1257–1266. 10.1111/jvs.12156.

[ece374306-bib-0065] Thuiller, W. , S. Lavorel , M. B. Araújo , and I. C. Prentice . 2005. “Climate Threats to Plant Diversity in Europe.” Proceedings of the National Academy of Sciences of the United States of America 102, no. 23: 8245–8250. 10.1073/pnas.0409902102. 15919825 PMC1140480

[ece374306-bib-0066] Tölgyesi, C. , M. Zalatnai , L. Erdős , Z. Bátori , N. R. Hupp , and L. Körmöczi . 2016. “Unexpected Ecotone Dynamics of a Sand Dune Vegetation Complex Following Water Table Decline.” Journal of Plant Ecology 9, no. 1: 40–50. 10.1093/jpe/rtv032.

[ece374306-bib-0067] Veblen, T. T. , and D. C. Lorenz . 1988. “Recent Vegetation Changes Along the Forest/Steppe Ecotone of Northern Patagonia.” Annals of the Association of American Geographers 78, no. 1: 93–111. 10.1111/j.1467-8306.1988.tb00193.x.

[ece374306-bib-0068] Vellend, M. 2010. “Conceptual Synthesis in Community Ecology.” Quarterly Review of Biology 85, no. 2: 183–206. 10.1086/652373. 20565040

[ece374306-bib-0069] Virtanen, R. , M. Luoto , T. Rämä , et al. 2010. “Recent Vegetation Changes at the High‐Latitude Tree Line Ecotone Are Controlled by Geomorphological Disturbance, Productivity and Diversity.” Global Ecology and Biogeography 19, no. 6: 810–821. 10.1111/j.1466-8238.2010.00570.x.

[ece374306-bib-0070] Willmott, C. J. , and K. Matsuura . 2005. “Advantages of the Mean Absolute Error (MAE) Over the Root Mean Square Error (RMSE) in Assessing Average Model Performance.” Climate Research 30, no. 1: 79–82. 10.3354/cr030079.

[ece374306-bib-0071] Wilson, E. O. 1992. The Diversity of Life. Harvard University Press.

[ece374306-bib-0072] Xu, L. , S. S. Chen , Y. Xu , G. Li , and W. Su . 2019. “Impacts of Land‐Use Change on Habitat Quality During 1985–2015 in the Taihu Lake Basin.” Sustainability 11, no. 13: 3513. 10.3390/su11133513.

[ece374306-bib-0073] Zeng, Y. , G. P. Malanson , and D. R. Butler . 2007. “Geomorphological Limits to Self‐Organization of Alpine Forest–Tundra Ecotone Vegetation.” Geomorphology 91, no. 3–4: 378–392. 10.1016/j.geomorph.2007.04.019.

