# Supplementary material for: Spatial Distribution and Ecological Assessment of Vegetation–Climate Zone Ecotones in the Temperate Forests of South Korea
Source: Ecol Evol. 2026 Sep 6;16(9):e74306. doi: 10.1002/ece3.74306 (PMC13546866; doi:10.1002/ece3.74306)
Supplement: Supplementary file 1 [file ECE3-16-e74306-s001.docx]

**Supplementary Material**

*Spatial distribution and ecological assessment of vegetation–climate zone ecotones in the temperate forests of South Korea*

**Table S1. Threshold-sensitivity analysis for ecotone zone (EZ) classification.**

| **Threshold (%)** | **No. of EZ grids** | **EZ proportion (%)** | **Shannon (EZ)** | **Shannon (core)** | **ΔShannon** | **Gini–Simpson (EZ)** | **Gini–Simpson (core)** | **ΔGini–Simpson** |
| --- | --- | --- | --- | --- | --- | --- | --- | --- |
| 50 | 71 | 8.9 | 0.811 | 0.3 | 0.51 | 0.647 | 0.234 | 0.413 |
| 55 | 109 | 13.6 | 0.767 | 0.279 | 0.488 | 0.621 | 0.215 | 0.406 |
| 60 | 166 | 20.8 | 0.718 | 0.248 | 0.471 | 0.591 | 0.187 | 0.404 |
| 65 | 201 | 25.1 | 0.696 | 0.228 | 0.468 | 0.576 | 0.168 | 0.408 |
| 70 | 228 | 28.5 | 0.681 | 0.212 | 0.469 | 0.564 | 0.154 | 0.41 |

*Note. Grids were classified as EZ when no single vegetation–climate zone exceeded the threshold proportion. ΔShannon and ΔGini–Simpson denote the mean difference in normalized entropy between EZ and core zones. The EZ/core separation (Δ values) remains stable across all thresholds, indicating that EZ classification is robust to threshold choice. Shannon entropy normalized by ln(4).*

**Table S2. Grid-size sensitivity analysis for EZ classification.**

| **Grid size (km)** | **Relative area (×)** | **No. of grids** | **No. of EZ grids** | **EZ proportion (%)** | **Shannon (EZ)** | **Shannon (core)** | **ΔShannon** |
| --- | --- | --- | --- | --- | --- | --- | --- |
| 5.5×6.5 | 0.25 | 3097 | 861 | 27.8 | 0.645 | 0.183 | 0.462 |
| 11×13 | 1.00 | 800 | 228 | 31.5 | 0.667 | 0.206 | 0.461 |
| 16.5×19.5 | 2.25 | 380 | 131 | 34.5 | 0.669 | 0.224 | 0.446 |
| 22×26 | 4.00 | 221 | 79 | 35.7 | 0.691 | 0.233 | 0.458 |

*Note. The vegetation–climate zone raster (250 m resolution) was re-aggregated at grid sizes ranging from 0.25× to 4× the original survey unit. The original 11×13 km grid (highlighted) — the standard unit of the national Nationwide Survey on Natural Environments — lies near the middle of the tested range. The EZ/core Shannon separation (ΔShannon) remains stable (0.446–0.462) across all grid sizes, indicating that EZ identification is robust to spatial resolution. Values are approximate (simple block re-aggregation) and differ slightly from the ArcGIS zonal-statistics values in the main text.*

**Table S3. Independent ecological validation of EZ classification using species data not used in the entropy-based delineation.**

| **Biodiversity metric** | **NTZ (n=60)** | **EZ (n=228)** | **CTZ (n=121)** | **STZ (n=128)** | **WTZ (n=263)** | **Kruskal–Wallis H** | **p** |
| --- | --- | --- | --- | --- | --- | --- | --- |
| Total species richness | 688.42 ± 20.31 | 607.59 ± 10.02 | 555.77 ± 13.70 | 483.00 ± 15.27 | 524.81 ± 12.83 | 73.76 | <0.001 |
| Endemic | 24.85 ± 1.12 | 13.39 ± 0.51 | 10.37 ± 0.55 | 8.21 ± 0.66 | 8.90 ± 0.35 | 176.39 | <0.001 |
| Endangered | 1.87 ± 0.21 | 0.78 ± 0.08 | 0.46 ± 0.09 | 0.76 ± 0.09 | 1.05 ± 0.09 | 58.81 | <0.001 |
| Alien | 64.68 ± 2.77 | 90.00 ± 1.68 | 88.70 ± 2.09 | 82.59 ± 3.13 | 82.73 ± 2.22 | 38.89 | <0.001 |

*Note. Values are mean ± SE species counts per grid cell. Zones are ordered along the temperature–altitude gradient (NTZ→EZ→CTZ→STZ→WTZ). EZ shows intermediate values for total richness, endemic, and endangered species, consistent with a transitional (ecotonal) position; alien species peak in EZ, indicating susceptibility to invasion (edge effect). Species were matched by Korean name to the national list of endemic (275/371 matched), alien (425/425), and endangered (52/52) species; unmatched Latin-name entries were excluded. EZ defined at the 70% threshold.*

**Table S4. InVEST model input data, sources, and parameter settings for the ecosystem-service indices (HQ, WY, SDR).**

**(a) Input datasets and sources**

| **Input variable** | **Dataset / product** | **Source** | **Reference period** | **Used for** |
| --- | --- | --- | --- | --- |
| Precipitation (annual mean) | WorldClim v2.1 Bioclimatic (BIO12), 30 arc-s | worldclim.org | 1970–2000 | HQ inputs / WY / SDR |
| Reference evapotranspiration | Terra MODIS 16A3GF.006 (500 m), via NASA AppEEARS | appeears.earthdatacloud.nasa.gov | 2000–2010 | WY |
| Root restricting layer depth | Detailed Soil Map (1:25,000), RDA Soil Portal | soil.rda.go.kr | 1964–1999 (digitized post-2000) | WY |
| Plant available water content | Detailed Soil Map (1:25,000), RDA Soil Portal | soil.rda.go.kr | 1964–1999 (digitized post-2000) | WY / SDR |
| Land use / land cover (LULC) | Sub-classified LULC map (mid-class), Env. Geographic Info. Service | egis.me.go.kr | 2019 imagery / 2020 compiled | HQ / WY / SDR |
| Digital elevation model (DEM) | SRTM (30 m) | NASA SRTM | — | elevation, slope, SDR (LS) |
| Threat layers (disaster, pollution) | Hazard-zone and pollution-source spatial data | National spatial data | — | HQ |

**(b) Habitat Quality — threat factors**

| **Threat** | **Max. distance (km)** | **Weight** | **Decay type** |
| --- | --- | --- | --- |
| Agriculture | 3 | 0.65 | linear |
| Mine | 6 | 0.48 | linear |
| Builtup | 8 | 0.9 | exponential |
| Road | 3 | 0.75 | linear |
| Railroad | 2 | 0.52 | linear |
| Pollution | 5 | 0.63 | exponential |
| Disaster | 2 | 0.4 | linear |

*Half-saturation constant (k) = 0.05 (model default).*

**(c) Habitat Quality — habitat suitability and sensitivity of each LULC class to threats**

| **LULC code** | **Habitat** | **Agriculture** | **Mine** | **Builtup** | **Road** | **Railroad** | **Pollution** | **Disaster** |
| --- | --- | --- | --- | --- | --- | --- | --- | --- |
| 110 | 0 | 0 | 0 | 0 | 0 | 0 | 0 | 0 |
| 120 | 0 | 0 | 0 | 0 | 0 | 0 | 0 | 0 |
| 130 | 0 | 0 | 0 | 0 | 0 | 0 | 0 | 0 |
| 140 | 0.1 | 0 | 0 | 0.1 | 0.1 | 0.1 | 0.1 | 0 |
| 150 | 0 | 0 | 0 | 0 | 0 | 0 | 0 | 0 |
| 160 | 0 | 0 | 0 | 0 | 0 | 0 | 0 | 0 |
| 210 | 0.3 | 0 | 0.65 | 0.69 | 0.59 | 0.55 | 0.6 | 0.55 |
| 220 | 0.45 | 0 | 0.61 | 0.75 | 0.61 | 0.57 | 0.64 | 0.59 |
| 230 | 0.2 | 0 | 0.43 | 0.46 | 0.39 | 0.37 | 0.4 | 0.37 |
| 240 | 0.4 | 0 | 0.63 | 0.72 | 0.6 | 0.56 | 0.62 | 0.57 |
| 250 | 0.3 | 0 | 0.65 | 0.69 | 0.59 | 0.55 | 0.6 | 0.55 |
| 310 | 0.9 | 0.7 | 0.72 | 0.9 | 0.78 | 0.75 | 0.75 | 0.65 |
| 320 | 1 | 0.7 | 0.72 | 0.9 | 0.78 | 0.75 | 0.75 | 0.65 |
| 330 | 0.85 | 0.7 | 0.72 | 0.9 | 0.78 | 0.75 | 0.75 | 0.65 |
| 410 | 0.8 | 0.67 | 0.68 | 0.75 | 0.7 | 0.65 | 0.6 | 0.5 |
| 420 | 0.35 | 0.5 | 0.51 | 0.56 | 0.53 | 0.49 | 0.45 | 0.38 |
| 510 | 0.8 | 0.75 | 0.68 | 0.85 | 0.78 | 0.75 | 0.75 | 0.65 |
| 520 | 0.4 | 0.3 | 0.2 | 0.5 | 0.42 | 0.4 | 0.7 | 0.6 |
| 610 | 0.75 | 0.85 | 0.87 | 0.98 | 0.77 | 0.74 | 0.87 | 0.67 |
| 620 | 0 | 0 | 0 | 0 | 0 | 0 | 0 | 0 |
| 710 | 0.75 | 0.7 | 0.75 | 0.9 | 0.64 | 0.62 | 0.8 | 0.6 |
| 720 | 1 | 0.82 | 0.85 | 0.96 | 0.75 | 0.72 | 0.85 | 0.35 |

*Habitat suitability: 0 = unsuitable, 1 = fully suitable. Sensitivity: 1 = highly sensitive, 0 = not affected. LULC codes: 100s urban/built, 200s agriculture, 300s forest, 400s grassland, 500s wetland, 600s barren, 700s water.*

**(d) Water Yield and Sediment Delivery — biophysical coefficients per LULC class**

| **LULC code** | **Root depth (mm)** | **Kc** | **LULC_veg** | **USLE_C** | **USLE_P** |
| --- | --- | --- | --- | --- | --- |
| 110 | 0 | 0.5 | 0 | 0 | 0 |
| 120 | 0 | 0.3 | 0 | 0 | 0 |
| 130 | 0 | 0.3 | 0 | 0 | 0 |
| 140 | 0 | 0.3 | 0 | 0 | 0 |
| 150 | 0 | 0.2 | 0 | 0 | 0 |
| 160 | 0 | 0.3 | 0 | 0 | 0 |
| 210 | 2000 | 0.65 | 1 | 0.1 | 0.12 |
| 220 | 2000 | 0.65 | 1 | 0.3 | 0.5 |
| 230 | 2000 | 0.7 | 1 | 0 | 0 |
| 240 | 3000 | 0.7 | 1 | 0.09 | 1 |
| 250 | 1000 | 0.85 | 1 | 0 | 0 |
| 310 | 7000 | 1 | 1 | 0.001 | 1 |
| 320 | 7000 | 1 | 1 | 0.001 | 1 |
| 330 | 7000 | 1 | 1 | 0.001 | 1 |
| 410 | 2000 | 0.65 | 1 | 0.15 | 1 |
| 420 | 2000 | 0.65 | 1 | 0.001 | 1 |
| 510 | 500 | 1 | 0 | 0 | 0 |
| 520 | 0 | 1 | 0 | 0 | 0 |
| 610 | 500 | 0.2 | 1 | 0 | 0 |
| 620 | 10 | 0.2 | 1 | 0 | 0 |
| 710 | 1000 | 1 | 0 | 0 | 0 |
| 720 | 0 | 1 | 0 | 0 | 0 |

*Root depth and Kc are Water Yield inputs; USLE_C and USLE_P are Sediment Delivery inputs. Rainfall erosivity (R), soil erodibility (K), and slope length–steepness (LS) were derived from WorldClim precipitation, the Detailed Soil Map, and the DEM, respectively. All values follow the standard national protocol (National Institute of Ecology 2022).*

**Table S5. Canonical correspondence analysis (CCA): partitioning of total and constrained inertia.**

Total inertia in species data: 7.6046. Constrained inertia (species–environment): 0.4884. Adjusted R² = 0.0548. Global permutation p = 0.001.

| **Canonical axis** | **Eigenvalue** | **% of total inertia** | **Cumulative % of total** | **% of constrained inertia** | **Spp–Envt Pearson r** |
| --- | --- | --- | --- | --- | --- |
| CCA1 | 0.211 | 2.8 | 2.8 | 57.2 | 0.966 |
| CCA2 | 0.108 | 1.4 | 4.2 | 29.3 | 0.887 |
| CCA3 | 0.05 | 0.7 | 4.9 | 13.6 | 0.806 |

*Note. Only three canonical (constrained) axes are extracted. The “% of constrained inertia” column corresponds to the values reported in the main text (CCA1 = 57.2%, CCA2 = 29.3%; cumulative 86.5%), i.e., the proportion of the species–environment (constrained) variation captured by each axis — not the proportion of total community variation. The low % of total inertia (first two axes = 4.2%) is expected for a large species matrix (4,712 species) and does not indicate a poor model; the first axis is statistically significant (permutation p = 0.001) and species–environment correlations are high (0.966, 0.887).*

**Table S6. Variance inflation factors (VIF) for the environmental variables used in CCA.**

| **Environmental variable** | **VIF** |
| --- | --- |
| Temperature | 4.63 |
| Precipitation | 2.74 |
| Slope | 5.63 |
| Elevation | 7.1 |
| Habitat quality (HQ) | 3.19 |
| Water yield (WY) | 2.5 |
| Sediment delivery ratio (SDR) | 4.08 |
| Bird diversity | 1.47 |

*Note. All VIF values are below 10 (maximum: Elevation = 7.10), indicating that multicollinearity does not invalidate interpretation of individual environmental vectors. Elevation and slope are moderately intercorrelated (r = 0.84) owing to natural topographic covariation and should be interpreted with corresponding caution.*
